# Convergent Metabolic Specialization through Distinct Evolutionary Paths in Pseudomonas aeruginosa

**DOI:** 10.1128/mBio.00269-18

**Published:** 2018-04-10

**Authors:** Ruggero La Rosa, Helle Krogh Johansen, Søren Molin

**Affiliations:** a Novo Nordisk Foundation Center for Biosustainability, Technical University of Denmark, Kgs. Lyngby, Denmark; b Department of Clinical Microbiology, Rigshospitalet, Copenhagen, Denmark; c Department of Clinical Medicine, Faculty of Health and Medical Sciences, University of Copenhagen, Copenhagen, Denmark; University of Washington

**Keywords:** adaptation, cystic fibrosis, environment, evolution, genomics, metabolism, metabolomics, specialization

## Abstract

Evolution by natural selection under complex and dynamic environmental conditions occurs through intricate and often counterintuitive trajectories affecting many genes and metabolic solutions. To study short- and long-term evolution of bacteria *in vivo*, we used the natural model system of cystic fibrosis (CF) infection. In this work, we investigated how and through which trajectories evolution of Pseudomonas aeruginosa occurs when migrating from the environment to the airways of CF patients, and specifically, we determined reduction of growth rate and metabolic specialization as signatures of adaptive evolution. We show that central metabolic pathways of three distinct Pseudomonas aeruginosa lineages coevolving within the same environment become restructured at the cost of versatility during long-term colonization. Cell physiology changes from naive to adapted phenotypes resulted in (i) alteration of growth potential that particularly converged to a slow-growth phenotype, (ii) alteration of nutritional requirements due to auxotrophy, (iii) tailored preference for carbon source assimilation from CF sputum, (iv) reduced arginine and pyruvate fermentation processes, and (v) increased oxygen requirements. Interestingly, although convergence was evidenced at the phenotypic level of metabolic specialization, comparative genomics disclosed diverse mutational patterns underlying the different evolutionary trajectories. Therefore, distinct combinations of genetic and regulatory changes converge to common metabolic adaptive trajectories leading to within-host metabolic specialization. This study gives new insight into bacterial metabolic evolution during long-term colonization of a new environmental niche.

## IMPORTANCE

Only a few examples of real-time evolutionary investigations in environments outside the laboratory are described in the scientific literature. Remembering that biological evolution, as it has progressed in nature, has not taken place in test tubes, it is not surprising that conclusions from our investigations of bacterial evolution in the CF model system are different from what has been concluded from laboratory experiments. The analysis presented here of the metabolic and regulatory driving forces leading to successful adaptation to a new environment provides an important insight into the role of metabolism and its regulatory mechanisms for successful adaptation of microorganisms in dynamic and complex environments. Understanding the trajectories of adaptation, as well as the mechanisms behind slow growth and rewiring of regulatory and metabolic networks, is a key element to understand the adaptive robustness and evolvability of bacteria in the process of increasing their *in vivo* fitness when conquering new territories.

## INTRODUCTION

Bacterial survival and replication during colonization of a new environment depend on sensing and responding to available nutrients and on activation of specific metabolic pathways which maximize growth efficiency (1). Accordingly, migration from one ecosystem to another initially challenges bacteria to respond with gene regulatory network plasticity and thereafter to develop metabolic specialization that can occur through loss of nonessential metabolic functions, through acquisition of metabolic genes, or by tailoring the expression and activity of metabolic pathways, which further increase the within-host fitness, maintenance of homeostasis, and support for coping with waste products and stresses (2, 3).

Pseudomonas aeruginosa is an environmental bacterium with broad metabolic versatility and a dynamic metabolic regulatory network that can support growth from a variety of nutritional resources. As an opportunistic pathogen, it frequently colonizes the airways of cystic fibrosis (CF) patients, where the nutrient composition and environmental conditions differ significantly from its normal natural habitat (4, 5). The CF airway environment is a multiniche structured environment with gradients of nutrients and oxygen, a complex multispecies microbiota, and numerous stress factors such as immune responses, antibiotics, and oxidative and osmotic stresses (6–8). Thus, the CF airways, in many respects, are similar to other natural environments confronting the invading bacteria with extraordinary challenges.

Migration of P. aeruginosa from the environment to the airways of CF patients implies reprogramming of the regulatory and metabolic networks according to the available nutrients and physicochemical conditions of the airways (1). Consequently, survival and adaptation depend on an equilibrium between robustness and evolvability (3, 9–13). Interestingly, it has been found that while environmental isolates of P. aeruginosa show high growth rates and diversified metabolism, airway-adapted isolates, in contrast, show reduced growth rates and a more specialized metabolism (14–17). While in the environment, macromolecule biosynthesis and growth rely on metabolite uptake or synthesis from the available nutrients; in contrast, during the infection, bacteria are restricted to the nutrient-rich environment of the CF sputum (5). Therefore, it has been hypothesized that long-term adaptation drives alterations of the metabolic repertoire to maximize growth at the cost of versatility. In many microorganisms such as Escherichia coli and Mycobacterium tuberculosis, reduced growth rates have previously been described to confer selective advantages under adverse environmental conditions and to facilitate tolerance to stresses such as the immune system and antibiotics (18).

However, in contrast to most *in vitro* adaptive laboratory evolution (ALE), how *in vivo* long-term evolution shapes the physiology of a bacterial organism and how it influences the balance between bacterial robustness and evolvability are still mostly unexplained. Discerning these biological questions is key to the understanding of how bacteria increase their fitness and how they become successful in conquering new environmental niches, where strong and continuous selective pressures drive evolution through complex fitness landscapes, which may affect many genes and metabolic solutions (6, 8, 19, 20).

Here, we describe how growth rate reduction and metabolic specialization develop over time in a case of three different P. aeruginosa lineages colonizing the lungs of a single CF patient. We have analyzed 26 clinical isolates of P. aeruginosa belonging to three different clone types, presenting naive, intermediate, and adapted phenotypes, sampled from a single CF patient over a period of 8 years of infection. Based on their genome sequences and metabolic abilities, we performed in-depth analyses of one early and one late isolate from each of the three clone types using time-resolved exometabolomics and correlation analysis of genomic and metabolic adaptive changes. This enabled us to characterize the trajectory of metabolic adaptation and to characterize the metabolic solution necessary for survival in the complex host environment of the CF lung. In contrast to previous studies that used indirect methodologies such as phenotypic microarrays (16, 21, 22), single-time-point metabolomics (23–25), or gene essentiality analysis (11) to evaluate the metabolic evolution of P. aeruginosa, our approach allowed us not only to obtain a direct readout of the cellular consumption and production rates of pathogenic isolates adapted to the complex environment but also to compare it to their mutational pattern. Finally, oxygen assimilation was assessed for the selected isolates, showing that within-host evolution changes the cellular oxygen requirements. Our integration of metabolomic and genomic data illustrates that within-patient evolution involved convergent metabolic specialization characterized by loss of nonessential metabolic functions and metabolic adaptation irrespective of the clone type, genomic composition, and mutation pattern.

## RESULTS

### Coexistence of Pseudomonas aeruginosa clone types in the airways of a CF patient.

**FIG 1:**
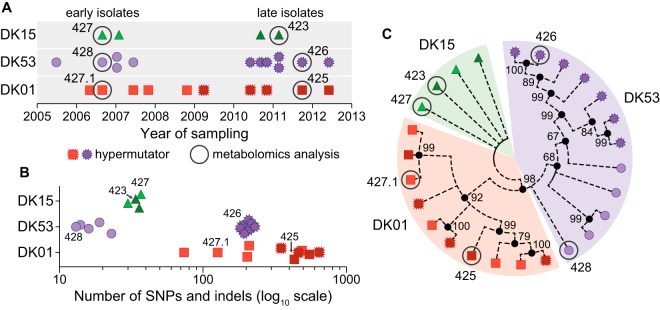
Genomic characterization of the longitudinal P. aeruginosa strain collection. (A) Year of isolation of the DK15 (green triangles), DK53 (purple circles), and DK01 (red squares) isolates sampled from patient P36F2. Light and dark shades for each clone type indicate early and late isolates, respectively. Jagged-edge symbols indicate hypermutator isolates. Metabolomics analysis was performed for the circled isolates, and their names are given. Detailed genomic features of all isolates are given in Table S1. (B) Total numbers of SNP and indel mutations detected in the clinical isolates. If multiple types of mutations were present in the same gene, the one with the highest functional impact was counted. The impact was ranked as low, intermediate, and high for silent, missense, or nonsense mutations, respectively. (C) Maximum parsimony reconstruction of the clinical isolates. The tree is based on 2,105 missense and nonsense SNP mutations that accumulated during within-patient evolution. Branches corresponding to partitions reproduced in less than 50% of bootstrap replicates are collapsed. The percentages of replicate trees in which the associated taxa clustered together in the bootstrap test (1,000 replicates) are shown next to the branches.

Despite extensive treatment with different antibiotic classes during 8 years of persistent infection, three distinct clone types (DK15, DK53, and DK01, assigned by sequence analysis as described in the work of Marvig et al. [19]) were repeatedly isolated from CF patient P36F2 attending the Copenhagen Cystic Fibrosis Center at the University Hospital, Rigshospitalet, Copenhagen, Denmark (Fig. 1A). The DK01 clone type is highly adapted and transmissible, being isolated from more than 40 CF patients after more than 40 years of within-patient evolution (26). The DK15 (naive) and DK53 (intermediate) clone types have a more recent evolutionary history, being isolated from only a few patients (19). MIC tests showed that many isolates from DK01 and a few from DK53 were resistant to antibiotics (see Fig. S1 in the supplemental material). The continued persistence of the susceptible bacteria may indicate collective resistance events, high tolerance to antibiotics, or treatment inefficiency during polyclonal infections.

10.1128/mBio.00269-18.2 FIG S1  Antibiotic sensitivity tests. MICs of the antibiotics that are indicated on top of each panel were determined for the isolates of the DK15 (green triangles), DK53 (purple circles), and DK01 (red squares) clone types. The dotted lines represent the EUCAST (version 7.1, 2017) clinical breakpoints. Download FIG S1, TIF file, 0.3 MB. Copyright © 2018 La Rosa et al. 2018 La Rosa et al. This content is distributed under the terms of the Creative Commons Attribution 4.0 International license.

10.1128/mBio.00269-18.9 TABLE S1  Mutations accumulated in Pseudomonas aeruginosa clinical isolates and distributions of mutated genes within the COG functional classes. Download TABLE S1, XLSX file, 0.5 MB. Copyright © 2018 La Rosa et al. 2018 La Rosa et al. This content is distributed under the terms of the Creative Commons Attribution 4.0 International license.

Single nucleotide polymorphisms (SNPs), as well as deletions and insertions (indels) relative to the reference strain PAO1, were previously identified in these isolates (19), ranging from tens (DK15 and early isolates of DK53) to hundreds (late isolates of DK53 and DK01) of mutations (Fig. 1B). During the infection, all late DK53 and 3 out of 5 late DK01 isolates evolved a hypermutator phenotype, due to nonsynonymous SNP mutations in the DNA mismatch repair genes *mutL* and *mutS*, respectively (Fig. 1A and B; Table S1) (21, 27). Maximum parsimony phylogenetic analysis on missense and nonsense SNP mutations clustered the clinical isolates into three distinct branches accordingly to their clone type (Fig. 1C), underlining the genetic distance within and between clone types.

### Reduction of metabolic abilities and growth rates during within-patient adaptation.

Long-term adaptation in a confined environment may occur by specialization involving changes in the bacterial physiology (9). We therefore tested the degree of adaptation of the clinical isolates by evaluating their metabolic versatility and growth potential.

**FIG 2:**
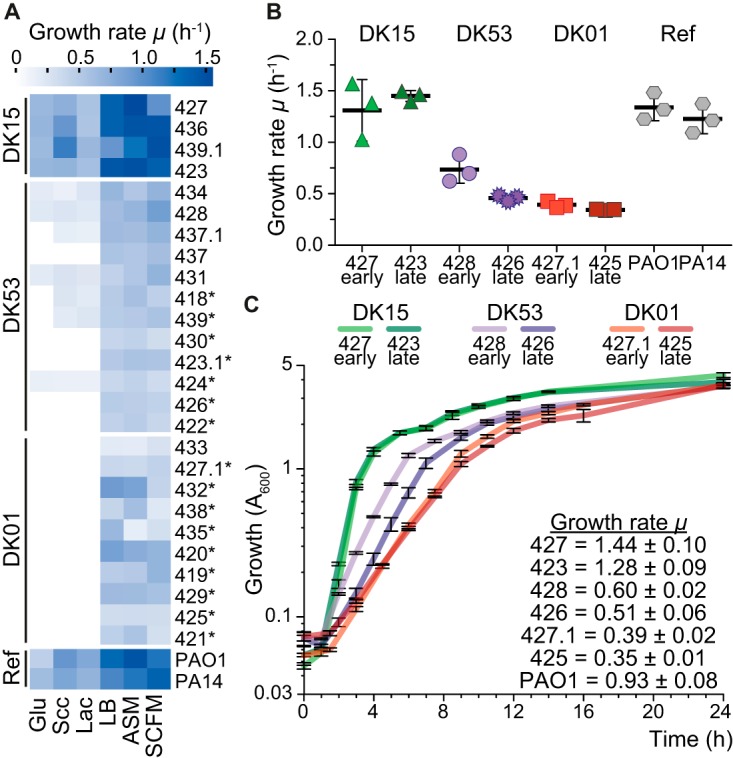
Reduced growth abilities of the P. aeruginosa clinical isolates. (A) Heat map of the specific growth rate (μ) in six different environments, including minimal medium supplemented with glucose (Glu), succinate (Scc), or lactate (Lac) and the rich medium LB, ASM (artificial sputum medium), or SCFM (synthetic CF sputum medium). Ref indicates the two reference strains PAO1 and PA14. Asterisks indicate isolates in which mutations of amino acid biosynthetic genes were identified that may explain the auxotrophy. Isolates are ordered by clone type followed by isolation date, early to late from top to bottom. (B) Cumulative growth rates in LB, ASM, and SCFM of the DK15, DK53, and DK01 clinical isolates used for the metabolomic analysis and of the reference strains PAO1 and PA14. Values represent the mean ± standard deviation of the average of the specific growth rate in each of the three media. Data are similar for all isolates within a clone type (Fig. S2A and C). (C) Growth profiles of DK15, DK53, and DK01 isolates in LB in batch cultures. Error bars represent the standard deviations (*n* = 3) and indicate the time points at which samples of culture supernatant were collected for dynamic exometabolome analysis. The growth rate values are given. The PAO1 growth profile is omitted due to overlap with DK15 isolates.

Growth of DK01 and most DK53 late isolates was impaired in minimal medium with either glycolytic or gluconeogenic carbon sources, presumably owing to auxotrophy (Fig. 2A) (14–16). In contrast, all isolates were able to grow in LB medium, artificial sputum medium (ASM), and synthetic CF sputum medium (SCFM) containing amino acids, organic acids, and sugars (Fig. 2A). Importantly, all DK01 and DK53 isolates showed reduced growth rates in all three rich media compared to both PAO1 and PA14 reference strains and to the DK15 isolates (one-way analysis of variance [ANOVA] followed by Tukey’s *post hoc* test; *P* < 0.0001) (Fig. 2A and S2A). The DK15 isolates showed, on average, 2.8-fold-higher growth rates than the DK01 isolates (Fig. 2B and C and S2A). In addition, the DK53 isolates showed a growth rate reduction across evolutionary time with early isolates growing faster than late ones (two-tailed unpaired *t* test, *n* = 15 to 21; *P* < 0.0001) (Fig. 2B and C and S2B). For each isolate, the cumulative growth rate values in the three rich media were similar (Fig. 2A and B and S2C), despite different compositions of LB medium, ASM, and SCFM.

10.1128/mBio.00269-18.3 FIG S2  Growth abilities of the clinical isolates in rich medium. (A) Cumulative growth rates in LB, ASM, and SCFM of the DK15, DK53, and DK01 isolates and the reference strains PAO1 and PA14 (Ref). Differences in growth rates were computed by one-way ANOVA followed by Tukey’s *post hoc* test (*n* = 6 to 36; *P* < 0.0001). (B) Cumulative growth rates in LB, ASM, and SCFM of the early and late isolates of DK53. Differences between the early and late isolates were computed by a two-tailed unpaired *t* test (*n* = 15 to 21; *P* < 0.0001). (C) Cumulative growth rates in LB, ASM, and SCFM of the clinical isolates and the reference strains PAO1 and PA14. Values represent the mean ± standard deviation of the average of the specific growth rate under each of the three conditions. Color codes for the clinical isolates are identical to those in the previous figures. Download FIG S2, TIF file, 0.3 MB. Copyright © 2018 La Rosa et al. 2018 La Rosa et al. This content is distributed under the terms of the Creative Commons Attribution 4.0 International license.

Interestingly, during within-patient evolution only the DK53 isolates changed their physiology. While the DK01 clone type embodied the characteristics of an adapted chronic pathogen (reduced metabolic abilities and growth rate), the DK15 clone type retained the characteristics of a naive colonizer (broad metabolic abilities and high growth rate). Therefore, although all isolates evolved within the same environment and were subject to the same stresses, evolution occurred dissimilarly.

### Metabolic specialization during within-patient adaptation.

It is known that the CF sputum is an amino-acid-rich substrate optimal for P. aeruginosa growth (5, 17) and that nutrient assimilation occurs in a sequential and tightly regulated manner with regard to both order and rate of assimilation (24). Therefore, we performed a dynamic exometabolome analysis of bacterial within-patient adaptation (i) to test whether it led to metabolic specialization and (ii) to deconvolute the metabolic trajectories. Based on our physiological data, we analyzed seven strains: two isolates (one early and one late) from each of the three DK15, DK53, and DK01 clone types covering 5 of the 8 years of within-patient adaptation and one from the reference strain PAO1 (Fig. 1A). Supernatants were collected during growth of each isolate in LB batch cultures (Fig. 2C) and subjected to targeted metabolomic analysis to identify amino acids, organic acids, and sugars. Metabolite measurements were performed on a total of 260 supernatant samples (Table S2).

10.1128/mBio.00269-18.10 TABLE S2  Raw data of the time course exometabolomics analysis of Pseudomonas aeruginosa clinical isolates and PAO1 reference strain. Download TABLE S2, XLSX file, 0.1 MB. Copyright © 2018 La Rosa et al. 2018 La Rosa et al. This content is distributed under the terms of the Creative Commons Attribution 4.0 International license.

**FIG 3:**
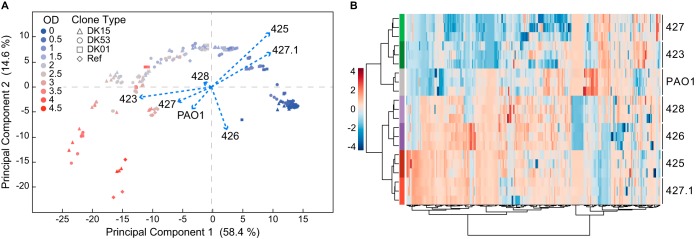
Relatedness of the exometabolomes of the P. aeruginosa clinical isolates. (A) Principal-component analysis (PCA) of the dynamic exometabolome profile of P. aeruginosa clinical isolates and the PAO1 reference strain. Each dot represents the metabolic status at a given OD. The isolates are projected as supplementary categorical classifiers to evaluate differences between the dynamic exometabolome profiles. Each biological replicate is plotted (*n* = 3). Together, PC1 and PC2 explained 73% of the variance in metabolite concentrations. (B) Hierarchical clustering of the dynamic exometabolome profiles of P. aeruginosa clinical isolates and the PAO1 reference strain. The profiles are clustered according to the Euclidean distance using the Ward clustering algorithm. Each biological replicate is plotted (*n* = 3).

To evaluate changes in the composition of the dynamic exometabolomes due to dissimilar assimilation and secretion of metabolites, we performed principal-component analysis (PCA) which shows the time-dependent trajectory of the metabolomes explaining 58.4% and 14.6% of the variance in PC1 and PC2, respectively (Fig. 3A and S3A). Even if similar trajectories of the metabolomes were displayed by all isolates, presentation of the isolates as categorical classifiers revealed a contrasting projection of the loadings of the two DK01 and the DK53 late isolates compared to the two DK15 isolates, PAO1, and the DK53 early isolate (Fig. 3A). Therefore, this strongly indicates that the identified compounds were differently metabolized by the isolates, supporting the hypothesis that within-host evolution resulted in metabolic specialization. PC1, therefore, represents metabolic patterns that explained evolution from the naive to an evolved stage, while PC2 explains the heterogeneity within the specific evolution stages. Similarly, when the PCA was scored after discarding the time variable from the analysis, the isolates grouped according to their clone type, with the DK15 isolates and PAO1 clustering together (Fig. S3). Hierarchical cluster analysis on metabolite concentration confirmed the previous results showing the relatedness between the DK15 isolates and PAO1 and between the DK01 and DK53 isolates, respectively (Fig. 3B).

10.1128/mBio.00269-18.4 FIG S3  Principal-component analysis (PCA) of the dynamic exometabolome profiles of P. aeruginosa clinical isolates and PAO1 as a reference strain. (A) Each dot represents the metabolic status at a given OD value, and the red arrows represent the loadings driving separation of the exometabolomes. The isolates are projected as supplementary categorical classifiers to evaluate differences between the dynamic exometabolome profiles. Each biological replicate is plotted (*n* = 3). Together, PC1 and PC2 explained 73% of the variance in metabolite concentrations. (B) The concentration of each metabolite over time was used as an independent variable. The three clone types and the reference strain were differentially clustered based on their metabolic fingerprint. Each biological replicate is plotted (*n* = 3). The ellipses represent the 95% confidence interval of the exometabolomes. Color codes for the clinical isolates are identical to those in the previous figures. Download FIG S3, TIF file, 0.3 MB. Copyright © 2018 La Rosa et al. 2018 La Rosa et al. This content is distributed under the terms of the Creative Commons Attribution 4.0 International license.

**FIG 4:**
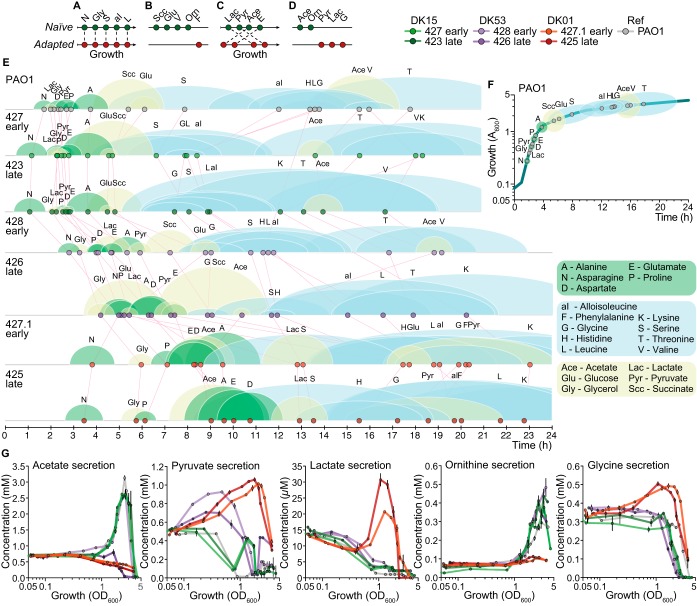
Dynamic assimilation and secretion of metabolites during growth of P. aeruginosa clinical isolates. (A to D) Categories of metabolites assimilated and secreted differently by the naive (DK15, DK53 early isolate, and PAO1 reference strain; green dots) and adapted (DK53 late isolate and DK01; red dots) isolates. Parallel plot of metabolites: assimilated with similar growth phase profiles in all isolates (A), not assimilated by all isolates (B), assimilated with changes in the growth phase profiles (C), and secreted differently (D). (E and F) Hierarchy of assimilation of the identified compounds of the clinical isolates and the PAO1 reference strain. (E) Parallel plot of the hierarchy of assimilation of the PAO1 reference strain and the clinical isolates. (F) Assimilation hierarchy of the PAO1 reference strain plotted onto the growth curve to facilitate the visualization of metabolic behaviors. The dots represent the *t*_50_ values (time at which the concentration of the metabolite decreased to 50% of the initial value) of the assimilated compounds. The circles (green for the amino acids assimilated during exponential phase and yellow for the organic acids and sugars) and ellipses (blue for the amino acids assimilated during stationary phase) represent the uptake windows (time needed to detect a reduction in the compound concentration from 75% to 25% of the initial value) of the assimilated compounds. (G) Organic acids acetate, pyruvate, and lactate and amino acids ornithine and glycine secreted transiently during growth. The curves represent the metabolite concentration (millimolar, except for lactate, which is micromolar) ± standard deviation (*n* = 3) relative to bacterial growth (OD_600_). DK15, DK53, and DK01 clinical isolates are represented by green, purple, and red circles, respectively. Light and dark shades for each clone type indicate early and late isolates, respectively.

We next mapped the metabolic behavior of the isolates modeling the hierarchy and rate of assimilation or secretion of metabolites by computing both their half-lives (*t*_50_ or *OD*_50_ values) and the assimilation times using a four-parameter nonlinear (sigmoid) model (28). A detailed representation of the assimilation map of the clinical isolates and the reference strain PAO1 is shown in Fig. 4E and F and S4. Analysis of the assimilation and secretion profiles revealed four categories of compounds, which characterized the metabolic trajectory of specialization from naive to adapted phenotype: (i) those assimilated with similar growth phase profiles in all isolates (asparagine, glycerol, serine, alloisoleucine, and leucine), representing conserved metabolic pathways independent of the evolutionary trajectory (Fig. 4A and E); (ii) those assimilated selectively by the naive isolates but not by the adapted ones (succinate, glucose, ornithine, and phenylalanine), indicating loss of nonessential metabolic function (Fig. 4B and E); (iii) those assimilated with changes in the growth phase profiles in the naive relative to the adapted isolates (lactate, pyruvate, acetate, and glutamate), indicating nutritional adaptation to the resources available in the CF sputum (Fig. 4C and E); and (iv) those secreted differently (acetate, ornithine, pyruvate, lactate, and glycine), indicating different distribution of the fluxes within the metabolic networks (Fig. 4D and G).

10.1128/mBio.00269-18.5 FIG S4  Assimilation parameters of the different compounds for the P. aeruginosa clinical isolates. The dots represent the *t*_50_ values (time at which the metabolite concentration decreased by 50% of the initial concentration) of the compounds assimilated by the PAO1 reference strain (Ref) and the clinical isolates with the dot colors as in the previous figures. The circles and ellipses show the assimilation time (time at which the metabolite concentration decreased from 75% to 25% of the initial concentration) of each compound. The color code is the same as in Fig. 4E. Download FIG S4, TIF file, 1 MB. Copyright © 2018 La Rosa et al. 2018 La Rosa et al. This content is distributed under the terms of the Creative Commons Attribution 4.0 International license.

**FIG 5:**
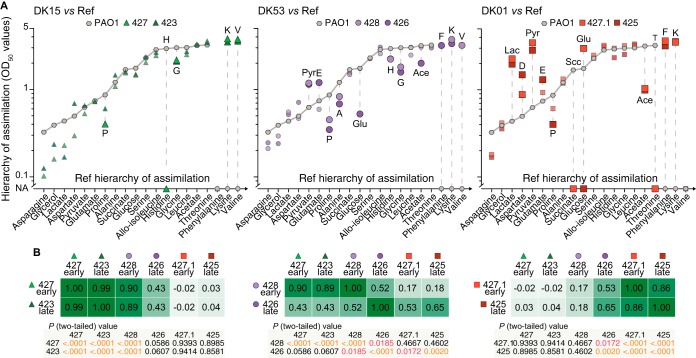
Changes in assimilation pattern during evolution. (A) Differences in the hierarchy of assimilation (*OD*_50_ values) of the clinical isolates DK15, DK53, and DK01 relative to PAO1 reference strain (gray line and hexagons). Cutoff values for the differences were set to higher than ±0.3 OD and ±0.6 OD during exponential and stationary phases, respectively. DK15, DK53, and DK01 clinical isolates are represented by green triangles, purple circles, and red squares, respectively. Light and dark shades for each clone type indicate early and late isolates, respectively. (B) Pearson correlation matrix for the *OD*_50_ values of the clinical isolates. The *r* coefficients and the *P* (two-tailed) values are indicated in the graph.

Comparing the metabolic behaviors of the strains, we found that the number of compounds with a different assimilation order in the clinical isolates from that in PAO1 increased with the progression of adaptation of the isolates. We considered *OD*_50_ differences compared to PAO1 of ±0.3 and ±0.6 of OD as biologically relevant for compounds assimilated during exponential and stationary phases, respectively. Differences ranged from four (DK15 isolate 423) to 11 (DK01 isolate 425) compounds (Fig. 5A). Interestingly, within the DK53 clone type, the number of the differentially assimilated compounds increased from seven to 11 for the early and late isolates during adaptation of this clone type (Fig. 5A). Moreover, the DK01 isolates showed the largest differences relative to DK15 and PAO1 in organic acid and sugar assimilation and secretion (Fig. 4 and S4). Pearson correlation analysis of the *OD*_50_ values showed consistency with the previous results (Fig. 3 and 5A), thereby confirming that there is no correlation between the DK15 and DK01 isolates (Pearson’s *r* = −0.02 to 0.03, respectively; 427.1 and 425, *P* [two-tailed] > 0.05) (Fig. 5B). The early DK53 isolate 428 strongly correlated with the DK15 isolates (Pearson’s *r* = 0.90 to 0.89, *P* [two-tailed] < 0.0001), while the late isolate 426 only moderately correlated with the DK15 and DK01 isolates (426 versus DK15 isolates, Pearson’s *r* = 0.43, *P* [two-tailed] = 0.0586 to 0.0607; 426 versus DK01 isolates, Pearson’s *r* = 0.53 to 0.65, *P* [two-tailed] = 0.0172 to 0.0020) (Fig. 5B). Changes in the assimilation or secretion pattern of one or more compounds in those categories, therefore, indicated that a transition from naive to adapted metabolism was occurring.

Altogether, these results confirm that metabolic specialization to the CF sputum occurred stepwise, through specific trajectories of metabolic evolution that converged to a distinctive metabolic behavior. During the infection, the DK53 isolates diverged in their metabolism from a pattern similar to the DK15 isolates (naive metabolism) toward one representative for the fully evolved DK01 isolates (adapted metabolism). Although we do not have access to early naive isolates of the DK01 clone type, the similarity between PAO1 and DK15 suggests that the phenotypic traits of DK01 were also initially similar to these other early isolates before eventually evolving to the present fully adapted stage.

### Metabolic adaptation to the CF sputum.

Since the metabolic changes that we observed may affect the nutritional preferences of P. aeruginosa in the lung environment, we analyzed the dynamic exometabolome data in the context of the CF sputum chemistry (5). As previously reported, compound utilization and secretion are influenced by the composition of the growth medium, most likely due to the different levels of carbon catabolite repression (29). However, comparison of the hierarchies of assimilation (*t*_50_ values) of P. aeruginosa PA14 grown in CF sputum and SCFM, as previously examined by Palmer and colleagues (5), and the reference strain PAO1 in LB medium showed positive correlation (Pearson’s *r* = 0.78 to 0.62, CF sputum and SCFM, respectively; *P* [two-tailed] > 0.05), even though the experimental setups and the methodological approaches were somewhat different. This strengthens the idea that our findings are relevant for growth in CF sputum.

The preferred compounds assimilated during early exponential phase by all the clinical isolates compared to PAO1 were asparagine and glycerol (Fig. 4A and E). However, these compounds have not been identified in CF sputum (5), emphasizing the need for a more comprehensive metabolomic analysis of the CF sputum to better understand the physiology and evolution of P. aeruginosa.

The growth phase profiles for the assimilation of compounds like serine, alloisoleucine, and leucine were similar irrespective of the isolate, thereby indicating conservation independent of the evolutionary trajectory in the host (Fig. 4A and E).

Succinate and glucose were assimilated during the transition phase and ornithine was assimilated during the stationary phase by all isolates except the DK01 ones (Fig. 4E and 5A). Since succinate is not present in the CF sputum and glucose is not a preferred compound in P. aeruginosa (30), these traits together with the reduced ability of adapted isolates to grow in the presence of gluconeogenic and glycolytic carbon sources (Fig. 2A) suggest that adaptation occurred through loss of nonessential metabolic functions similarly to what was described previously (31).

Phenylalanine, lysine, and valine are abundant amino acids in CF sputum (5), but their assimilation occurred with clone-type-specific differences, at a low rate, and only during late stationary phase (Fig. 4E and 5A). These carbon sources are therefore less preferred and may be important only when other carbon sources become limited or exhausted.

In the lungs of CF patients, oxygen availability fluctuates substantially, thereby contributing to both niche colonization and metabolic specialization (32, 33). Moreover, abundant nitrate (NO_3_^−^) and nitrite (NO_2_^−^) have been found in CF secretions (34, 35). In the absence of both O_2_ and nitrogen oxides, P. aeruginosa can ferment pyruvate and arginine, producing acetate and producing lactate and ornithine (36), respectively, for long-term survival and redox balance rather than growth. During stationary phase, we detected large amounts of secreted acetate (~2.5 mM) for PAO1 and the two DK15 and the early DK53 isolates and secreted ornithine (~0.5 mM) for both DK15 and DK53 isolates and PAO1. In contrast, the late DK53 and the two DK01 isolates assimilated acetate during both exponential and stationary phases (Fig. 4G). During growth, DK53 and DK01 isolates first secreted pyruvate, which was then assimilated (Fig. 4G). During late exponential phase, the DK01 isolates also secreted small amounts of lactate and glycine (Fig. 4G). These results are in agreement with previous studies showing that acetate production negatively correlates with the length of infection (24) and confirm that within-host adaptation is associated with different distributions of metabolic fluxes and loss of nonessential metabolic function, leading to reduced fermentation processes. This result is also supported by the fact that in sputum samples only 15% of the cells are in the stationary phase of growth (17).

Interestingly, the compounds that were assimilated during exponential growth of all clone types mainly fed the tricarboxylic acid (TCA) cycle, with DK01 assimilating 30 to 40% fewer carbon sources than PAO1 (Fig. S5). This may explain, at least partially, why DK01 isolates showed reduced growth rates. Since our analysis focused on amino acids, organic acids, and sugars, we cannot exclude that the DK01 isolates assimilated di- and tripeptides or fatty acids that can be present in LB or CF sputum.

10.1128/mBio.00269-18.6 FIG S5  Pathways involved in the assimilation of the compounds during exponential phase. Levels of green represent the hierarchy of assimilation. Blue arrows show nonassimilated compounds. The thickness of the arrows represents the ratio of the assimilation time for the clinical isolate to that for the reference strain PAO1. Dashed lines represent multistep assimilation pathways. Download FIG S5, TIF file, 0.7 MB. Copyright © 2018 La Rosa et al. 2018 La Rosa et al. This content is distributed under the terms of the Creative Commons Attribution 4.0 International license.

### Increase in oxygen consumption during within-patient adaptation.

Since fermentation was favored in the DK15 and early DK53 isolates (Fig. 4G), we hypothesized that O_2_ was differentially consumed in the three clone types. P. aeruginosa can grow aerobically or anaerobically by performing either respiration, by using a broad set of high- and low-affinity O_2_ terminal oxidases as terminal electron acceptors and nitrogen oxides for denitrification, or fermentation (36, 37). Therefore, differential O_2_ consumption may have important implications for the adaptive state of the isolates.

**FIG 6:**
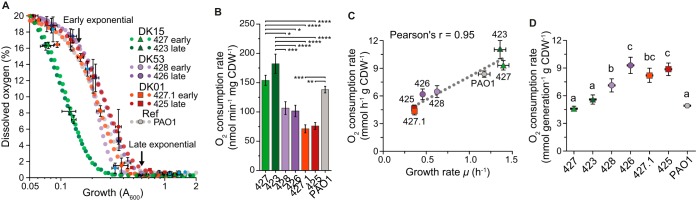
Efficiency of oxygen consumption in evolved clinical isolates. (A) Measurements of dissolved oxygen levels during batch cultures, fitted to a four-parameter sigmoid model. Error bars represent standard errors (*n* = 4). Early and late exponential phases are indicated with arrows. (B) Oxygen consumption rate during exponential phase. The values represent the mean for the rates at ODs of 0.15 and 0.6 ± standard error (*n* = 8). Specific rates at ODs of 0.15 and 0.6 are presented in Fig. S6A. Differences between isolates were computed using the one-way ANOVA followed by Tukey’s *post hoc* test (*P* values: ****, <0.0001; ***, <0.001; **, <0.01; *, <0.05). CDW, dry weight of cells. (C) Linear correlation between oxygen consumption and growth rates. The significance was determined by Pearson correlation test (*r* = 0.95, *P* [two-tailed] = 0.0008). (D) The level of oxygen assimilated per generation was calculated as the amount of oxygen consumed per duplication during exponential phase. Values correspond to means ± standard errors. Differences between isolates were computed using the one-way ANOVA followed by Tukey’s *post hoc* test. Different letters indicate statistically significant differences between the means (in all cases, *P* is <0.0001, except 423 versus 428, for which *P* is <0.001). Color codes for the clinical isolates are identical to those in the previous figures.

When assessing O_2_ consumption in batch cultures, we found that dissolved oxygen was exhausted at OD values from 0.2 for DK15 isolates to 0.5 for DK01 isolates (Fig. 6A). During exponential phase, the rate of O_2_ consumption was significantly higher in DK15 cultures than in DK53 and DK01 cultures (Fig. 6B and S6A). Although the Pearson correlation showed that oxygen consumption and growth rates were strongly related (*r* = 0.95, *P* [two-tailed] = 0.0008) (Fig. 6C), oxygen consumption per generation was 1.5- to 2-fold higher in DK01 than in DK15 isolates, with DK53 isolates showing intermediate values (Fig. 6D). Growth of the isolates in rich medium with defined oxygen levels as the limiting factor showed that the DK01 isolate and the late DK53 isolate reached a final OD about half of that of the DK15 isolates (Fig. S6B), therefore confirming higher oxygen consumption for the adapted isolates.

10.1128/mBio.00269-18.7 FIG S6  Oxygen consumption and final OD at limiting oxygen concentration. (A) Oxygen consumption rate during early and late exponential phases. The values represent means of the rate at ODs of 0.15 and 0.6 ± standard error (*n* = 4). Differences between the isolates were calculated using one-way ANOVA (*P* values: ****, <0.0001; ***, <0.001; **, <0.01; *, <0.05). (B) OD reached after 24 h of growth in the rich media LB, ASM, and SCFM at limiting oxygen concentration. Differences between the isolates were calculated using one-way ANOVA followed by Tukey’s *post hoc* test (*P* values: ****, <0.0001; ***, <0.001; **, <0.01; *, <0.05). Color codes for the clinical isolates are identical to those in the previous figures. Download FIG S6, TIF file, 0.4 MB. Copyright © 2018 La Rosa et al. 2018 La Rosa et al. This content is distributed under the terms of the Creative Commons Attribution 4.0 International license.

These results confirm that oxygen metabolism was a target of adaptive evolution and suggest that in an environment with reduced oxygen availability, a high oxygen requirement may result in limiting biomass accumulation. Moreover, as shown previously, the DK53 isolates diverged from a naive metabolic stage similar to that of the DK15 clone type to converge to the adapted one of the DK01 clone type.

### Relationship between metabolic and genomic changes.

Although 1,667 mutations were identified in metabolic genes in the clinical isolates (Fig. S7; Table S1), it was not obvious from scrutinizing the genome sequences how they influenced the metabolic behavior. Functional grouping of the genes with missense and nonsense mutations revealed statistical enrichment, although at very low significance, of those involved in metabolic processes, including amino acid transport and metabolism (1.3-fold), defense mechanisms (1.4-fold), signal transduction mechanisms (1.4-fold), and translation (0.6-fold) (Fig. S7A; Table S1). Moreover, only a few genes altered by mutations were shared within and between clone types (Fig. S7B), indicating limited parallel evolution. Interestingly, the number of missense and nonsense SNPs and indels during the evolution of DK01 clone type increased from 90 (isolate 427.1) to 298 (isolate 425) (Fig. 1B; Table S1), of which only 34 were shared, without any detectable metabolic changes.

10.1128/mBio.00269-18.8 FIG S7  Characterization of SNP and indel mutations. (A) Number of genes, grouped by functions, that contain missense and nonsense SNPs and indel mutations and enrichment of genes mutated relative to expectance. Silent and intergenic mutations were not taken into account. The genes were grouped based on the Clusters of Orthologous Groups (COGs) of Proteins database (59). A detailed list of mutations is given in Table S1. Asterisks denote functional classes that are significantly overrepresented relative to expectance. (B) Venn diagram of the number of genes mutated in more than half of the isolates of each clone type. Download FIG S7, TIF file, 0.5 MB. Copyright © 2018 La Rosa et al. 2018 La Rosa et al. This content is distributed under the terms of the Creative Commons Attribution 4.0 International license.

Therefore, to both estimate the genotype-to-phenotype correspondence and ascribe the observed metabolic changes to specific genomic changes, we investigated the presence of missense and nonsense mutations affecting metabolic pathways, transporters, and regulatory networks involved in the assimilation and secretion of some of the identified metabolites. Surprisingly, correspondence could be identified in only one case, i.e., the hypothesized auxotrophic traits observed in DK01 and in five DK53 isolates during growth in the presence of succinate, glucose, or lactate as sole carbon source (Fig. 2A). Indeed, at least one nonsynonymous mutation in amino acid biosynthetic genes was identified in 9 of 10 and 7 of 12 isolates of the DK01 and DK53 clone types, respectively (Fig. 2A; Table S1). Of the 33 identified amino acid biosynthetic genes containing nonsynonymous mutations, an overrepresentation test showed that methionine, leucine, tryptophan, arginine, histidine, and lysine biosynthetic pathways were more often altered by mutations (Table S1).

No mutation was found in the known genetic determinants for the glucose and succinate catabolic pathways, suggesting that the inability of the adapted isolates to assimilate them might be related to regulatory changes (Fig. 4B and E; Table S1). The lack of valine assimilation that occurred in the DK01 isolates might be explained by a nonsense mutation in the branched-chain amino acid transporter gene *braF* (Fig. 4B and E; Table S1) (38). The DK01 isolates assimilated lactate only during stationary phase due to its transient secretion during late exponential phase (Fig. 4G). However, we found a nonsynonymous mutation in the lactate permease *lldP* gene in isolate 425 (39) (Table S1), which suggests that lactate may have been assimilated through other unspecific transporters or that the mutation does not affect the function.

Under anaerobic conditions, arginine fermentation into ornithine involves the *arcDABC* operon (40, 41). The DK01 and DK53 isolates did not secrete ornithine, and we found no mutations in the genes of the corresponding pathway (Fig. 4G; Table S1). Instead, we found a missense mutation in the early DK01 isolate 427.1 in *oruR*, a regulator-encoding gene whose deletion was shown to cause the loss of ornithine assimilation (42). Anaerobic fermentation of pyruvate to acetyl coenzyme A (acetyl-CoA) and further to lactate and acetate involves the *aceEF*, *ldhA*, and *ackA-pta* genes, respectively (43). While no mutations were found in these genes in the DK01 isolate, a nonsynonymous and a synonymous mutation were found in the late DK53 isolate 428 in *ackA* and *pta*, respectively, that may explain reduced acetate production (Fig. 4G; Table S1). These results indicate that reduced fermentation mostly occurred owing to differential distribution of fluxes and/or regulatory changes rather than to mutations in specific metabolic pathways.

In P. aeruginosa, carbon catabolite repression (CCR) regulates the preference and hierarchy of carbon source assimilation through the activity of the CbrA/CbrB two-component system and the Crc-Hfq-CrZ regulatory system that involves an intricate repression network of transcriptional regulators, transporters, and metabolic enzymes (28, 30). Since we found very few mutations that could explain the observed changes (Fig. 4E and 5A), the metabolic specialization of the adapted DK01 and late DK53 isolates might depend on the tailored activity of the CCR system, which during evolution narrows the preference and hierarchy of assimilation to the nutritional resource present in the CF sputum, or on regulatory network changes that modify the expression and activity of metabolic pathways and enzymes.

Lower oxygen consumption rates and higher nitrogen oxide utilization have been previously described in *lasR* mutant clinical isolates (44). Here, we detected no mutations in *lasR* (Table S1). However, the DK01 isolates carried mutations in *cyoB* and *cioA* encoding terminal oxidases that may explain decreased O_2_ consumption rates (Fig. 6).

In summary, we show that adaptive processes converged to similar phenotypes in the DK01 and DK53 lineages through metabolic rewiring. Furthermore, diverse changes in both regulatory and metabolic networks enabled genetically distinct colonizing strains to follow the same adaptive phenotypic trajectory and achieve persistence in the lungs.

## DISCUSSION

Characterizing the adaptive trajectories during within-patient evolution is pivotal to understanding host-pathogen interactions and to understanding the adaptive evolution mechanisms occurring *in vivo*. P. aeruginosa metabolism is very diverse, and a detailed knowledge of how the evolution of metabolism supports infection factors such as mucoidity, biofilm formation, motility, virulence, and pathogenicity is still lacking.

**FIG 7:**
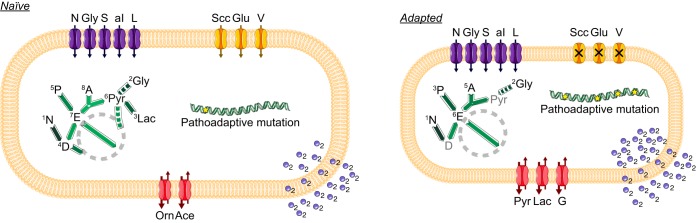
Model of the metabolic specialization of naive and adapted P. aeruginosa clinical isolates. While the naive isolates show high growth rate, broad metabolic versatility, hierarchy of assimilation tailored to the environment, high fermentation processes, low oxygen requirement, and limited number of mutations, during within-patient evolution the adapted isolates reduced their growth rate, lost metabolic versatility, tailored the hierarchy of assimilation of the nutrients to the CF sputum, reduced the fermentation processes, increased the oxygen requirement, and accumulated a large number of pathoadaptive mutations. The reduced growth rate of adapted isolates is represented by a smaller cell size. Violet, orange, and red shapes represent the transporters for those metabolites for which assimilation is conserved independently of the stage of adaptation, for those for which assimilation occurs only in the naive isolates, and for those for which secretion occurs differentially in the different isolates, respectively. The green arrows represent the hierarchy of assimilation of the nutrients and the distinct distribution of the metabolic fluxes in the metabolic pathways. The blue circles indicate the oxygen requirement of the cell, and the pathoadaptive mutations accumulated in the genome are represented by the yellow stars.

Here, we present a high-resolution metabolic footprinting of clinical isolates of the opportunistic pathogen P. aeruginosa over 8 years of adaptive evolution in a complex dynamic niche, which we correlated with the genome sequences and mutational patterns from a prior genomic study (19). Our study involved a dynamic survey of a longitudinal collection of isolates of three distinct clone types (DK01, DK15, and DK53) at various stages of evolution during adaptation to the lungs of a CF patient. We compared evolutionary trajectories within the same environment and selective pressures and showed that, even if evolution in the host converged, it occurred dissimilarly. While the DK15 and DK01 isolates maintained their initial phenotypes, within-patient evolution of the DK53 isolates was characterized by ongoing metabolic specialization. Altogether, our results show that evolution converged toward the loss of nonessential metabolic functions, metabolic simplification, and metabolic specialization. These major physiological changes, summarized in Fig. 7, were acquired through distinct genomic evolutionary trajectories and mutational patterns converging to similar phenotypes. The evolved isolates, adapting to the nutritional resources present in the CF sputum (5) and the environmental conditions, displayed (i) reduced growth rates; (ii) preferential assimilation of amino acids with a specific nutritional hierarchy designed for the CF lung resources, thereby limiting their biosynthesis; (iii) reduced fermentation processes; and (iv) an increased oxygen requirement.

The CF sputum is a complex and viscous substrate rich in amino acids, DNA, lipids, and mucin, and the composition can vary from patient to patient (4). A synthetic formulation (SCFM) resembling its natural composition was designed after analyzing the CF sputum from 12 patients (5). However, even though the SCFM has been useful to simulate the *in vivo* CF nutrient composition and it is used by many researchers, it is still challenging to mimic the CF sputum and the lung environment *in vitro*. Indeed, although clinical isolates grow in SCFM in sealed microtiter plates, neither DK53 nor DK01 isolates could grow in Erlenmeyer flasks, which were required for the metabolomics analysis. This difference in growth ability might be caused by limitation of oxygen (45), which may develop during within-patient evolution, but further investigations are necessary to confirm this hypothesis. Therefore, to overcome this limitation, and taking into account that our study intended to compare the metabolic behaviors of the clinical isolates beyond the *in vivo* CF environment, we decided to use the LB medium, which also contains amino acids and sugars although at different concentrations than in SCFM.

One of the major environmental selective forces in the CF lung is the continuous presence of antibiotics. Our results are in agreement with the metabolic evolution of antibiotic resistance observed in an *in vitro* adaptive laboratory evolution (ALE) experiment in Escherichia coli where replicate population lineages evolving under antibiotic selective pressures converged to highly similar metabolic steady states, which constrained the bacterial evolutionary trajectories (46). However, while ALE experiments typically select for fast-growing cells (47, 48), the long-term *in vivo* evolution setting within CF patients revealed enrichment for slow-growing isolates (17). This indicates that slow-growth selection is not directly coupled to antibiotic presence. Reduced growth rates have been described for E. coli, Streptococcus pneumoniae, Staphylococcus aureus, and M. tuberculosis to favor tolerance to antibiotics (18). Whether slow-growing isolates of P. aeruginosa also exhibit higher levels of tolerance to antibiotics remains to be shown.

In most *in vitro* ALE experiments, including the longest-running one (49), it is often difficult to draw precise genotype-to-phenotype correlations and causality maps, mostly due to beneficial mutations in genes with complex pleiotropic effects (50–54). In the much more complex and continuously changing environment of infected CF patients, it is even more difficult to correlate mutations with phenotypic traits. Only in the cases of auxotrophic traits did we identify causal mutations. P. aeruginosa has a large genome (55) characterized by redundancy, duplications of metabolic enzyme-encoding genes, the presence of paralogous metabolic pathways, many transcriptional regulators, and many genes of unknown function. Moreover, distinct clone types usually acquire different combinations of pathoadaptive mutations (19, 56, 57), which in turn produce genetically heterogeneous bacterial communities in coinfecting scenarios. Although this study includes isolates from only one case (CF patient), several phenotypes associated with each of the three lineages, such as change of growth rate, auxotrophy, development of antibiotic resistance, and hypermutability, have previously been ascribed to different P. aeruginosa isolates from random patients (7, 14, 17, 27, 58). The finding that similar characteristics are observed over time of infection in the studied environment (patient) strengthens our conclusions concerning the metabolic specialization occurring in the host. The adaptive processes described for DK01, DK53, and DK15 clone types are, therefore, representative, even though the actual trajectories may vary from strain to strain and from patient to patient. Despite this heterogeneity, our study suggests that convergent metabolic specialization can be a signature of adaptive evolution after conquering a new niche through a combination of mechanisms, including mutations, transcriptional and translational regulation, and epistasis.

## MATERIALS AND METHODS

### Bacterial strains and media.

P. aeruginosa clinical isolates are listed in Table S1 in the supplemental material and were sampled and identified from sputum samples as previously described elsewhere (19). Analyses of the bacterial isolates from patient P36F2 attending the Copenhagen Cystic Fibrosis Center at the University Hospital, Rigshospitalet, Copenhagen, Denmark, were approved by the local ethics committee of the Capital Region of Denmark (Region Hovedstaden; registration number H-1-2013-032). Growth was assessed in LB medium and M9 minimal medium with glucose, succinate, or lactate.

### Time-resolved exometabolome analysis and quantification of amino acids, organic acids, and sugars.

Supernatant samples were collected for isolates 427, 423, 428, 426, 427.1, and 425 and for the reference strain PAO1 over 24 h of growth. Quantification of amino acids was performed using the EZ:faast amino acid analysis kit (Phenomenex, USA) according to the provided protocol. Organic acids and sugars were quantified with a high-pressure liquid chromatography system coupled to a variable wavelength and refractive index detector (HPLC-UV-RI; Ultimate 3000; Dionex, USA).

### Measurement of oxygen consumption.

Decrease in oxygen saturation was measured during growth using a Unisense (Aarhus, Denmark) Clark-type oxygen sensor according to the manufacturer’s instructions.

### Comparative genomics and statistical analyses.

Genomic data consisting of SNPs and indels are available for the different isolates (19). Maximum parsimony (MP) analysis was conducted in MEGA7 version 7.0.26. Principal-component analysis (PCA) and hierarchical cluster analysis were performed using the software JMP version 13.0. Growth rates, Pearson correlations, and functional enrichments were scored using the software GraphPad Prism version 7.0a.

### Additional information.

Additional details regarding our experimental procedures and materials are provided in Text S1 in the supplemental material.

10.1128/mBio.00269-18.1 TEXT S1  Supplemental materials and methods. Download TEXT S1, PDF file, 0.1 MB. Copyright © 2018 La Rosa et al. 2018 La Rosa et al. This content is distributed under the terms of the Creative Commons Attribution 4.0 International license.
