# Supplementary figures and images for: Convergent Metabolic Specialization through Distinct Evolutionary Paths in Pseudomonas aeruginosa
Source: mBio. 2018 Apr 10;9(2):e00269-18. doi: 10.1128/mBio.00269-18 (PMC5893872; doi:10.1128/mBio.00269-18)

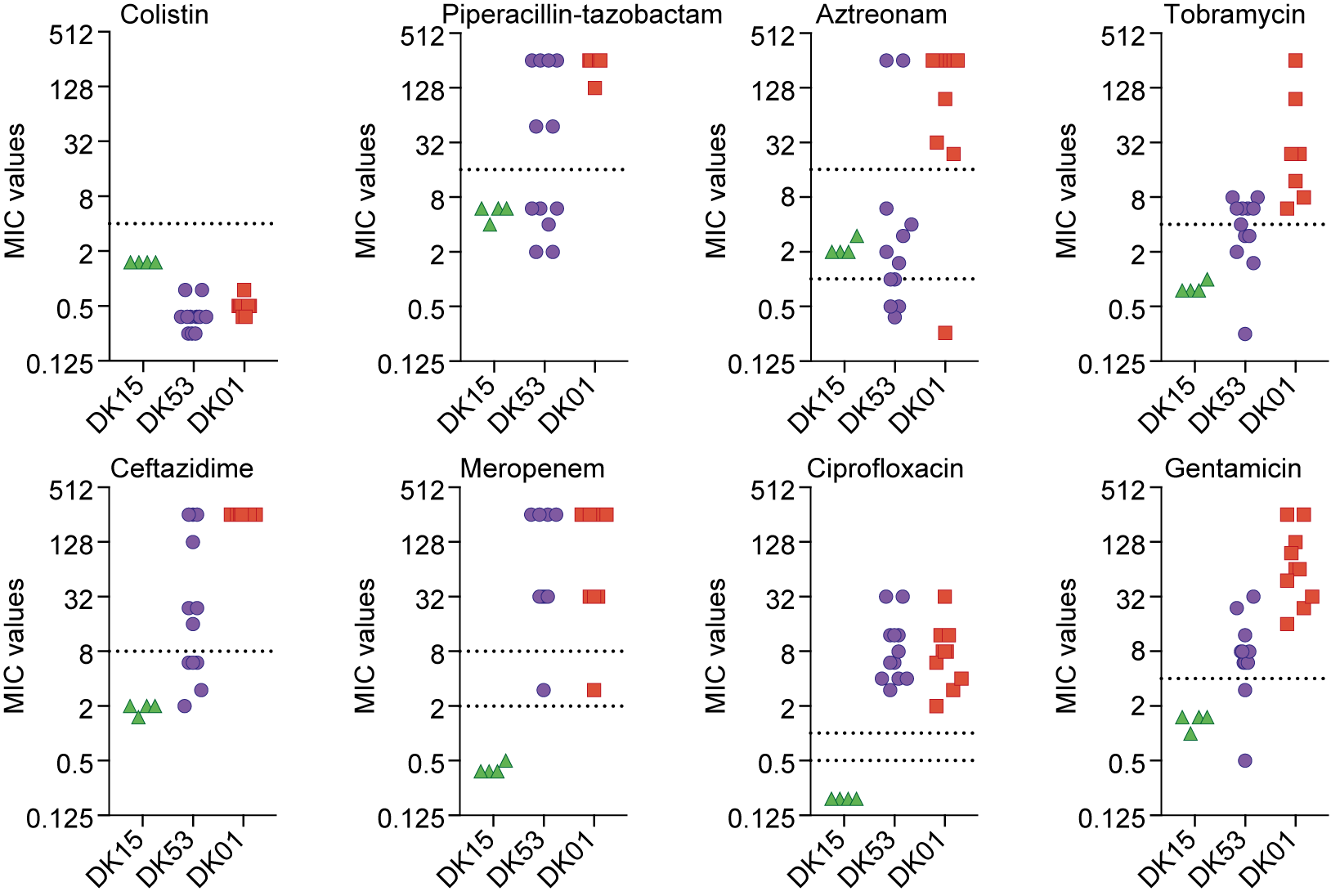

Supplement: FIG S1 [file mbo001183819sf1.tif]

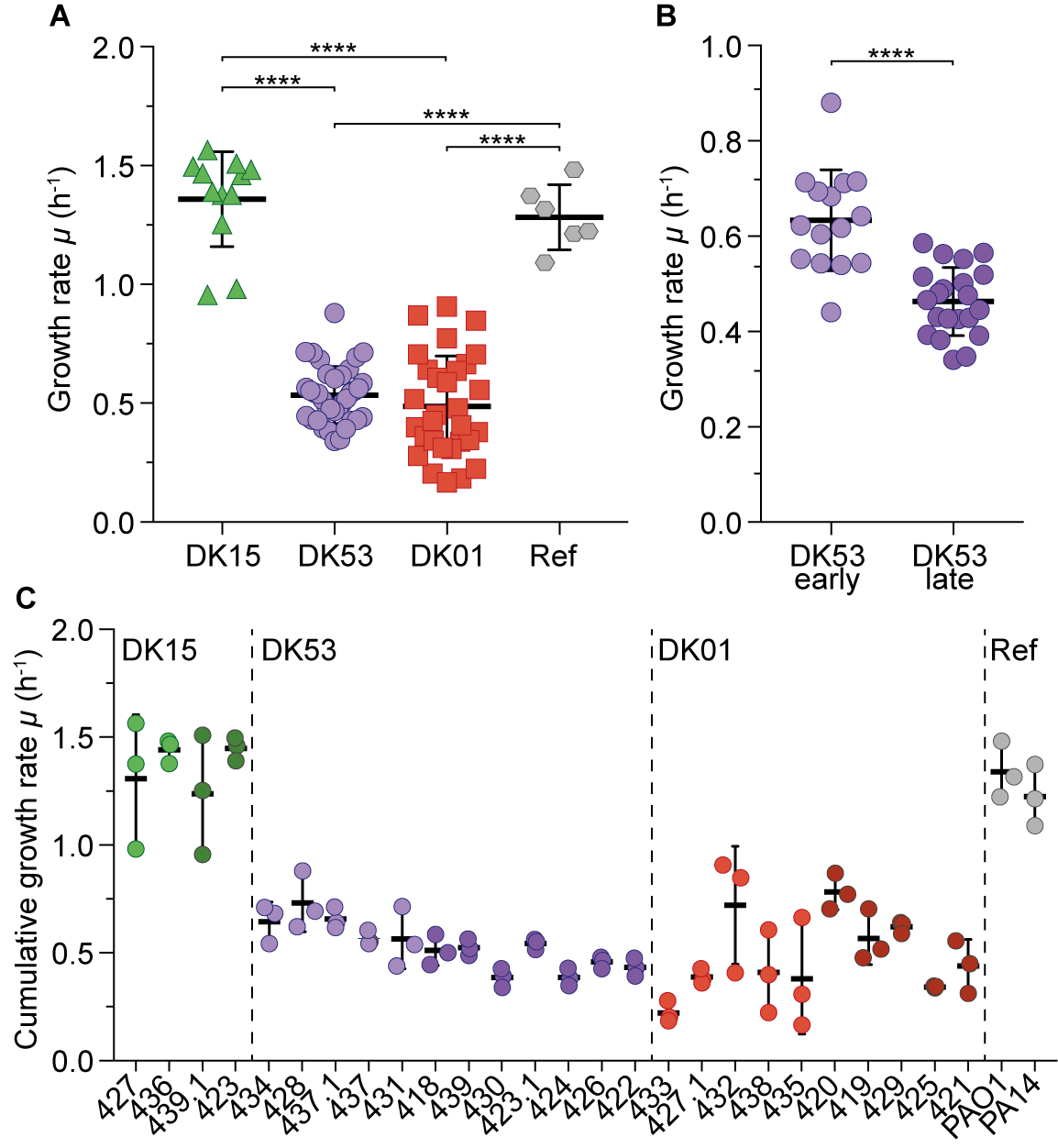

Supplement: FIG S2 [file mbo001183819sf2.tif]

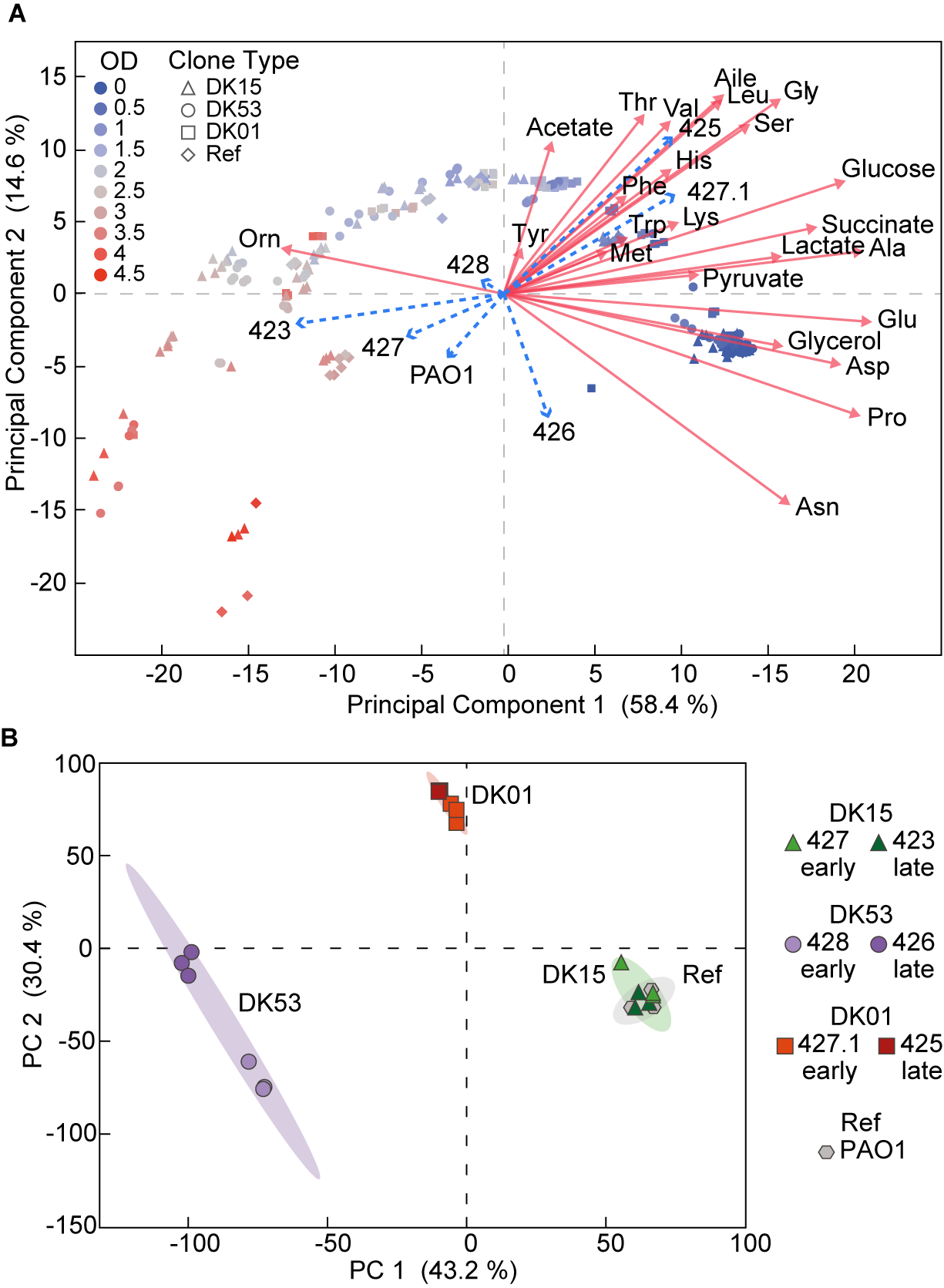

Supplement: FIG S3 [file mbo001183819sf3.tif]

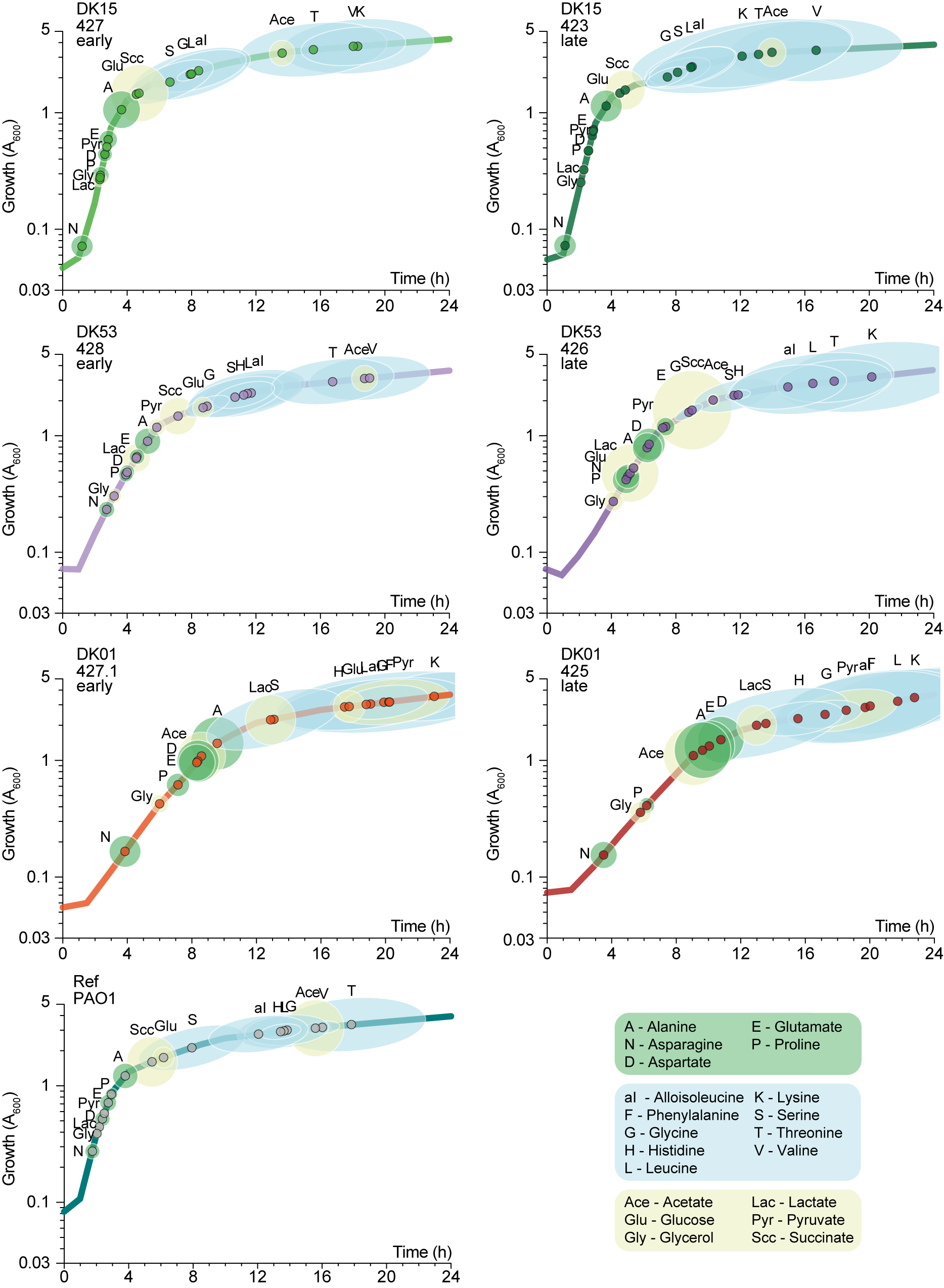

Supplement: FIG S4 [file mbo001183819sf4.tif]

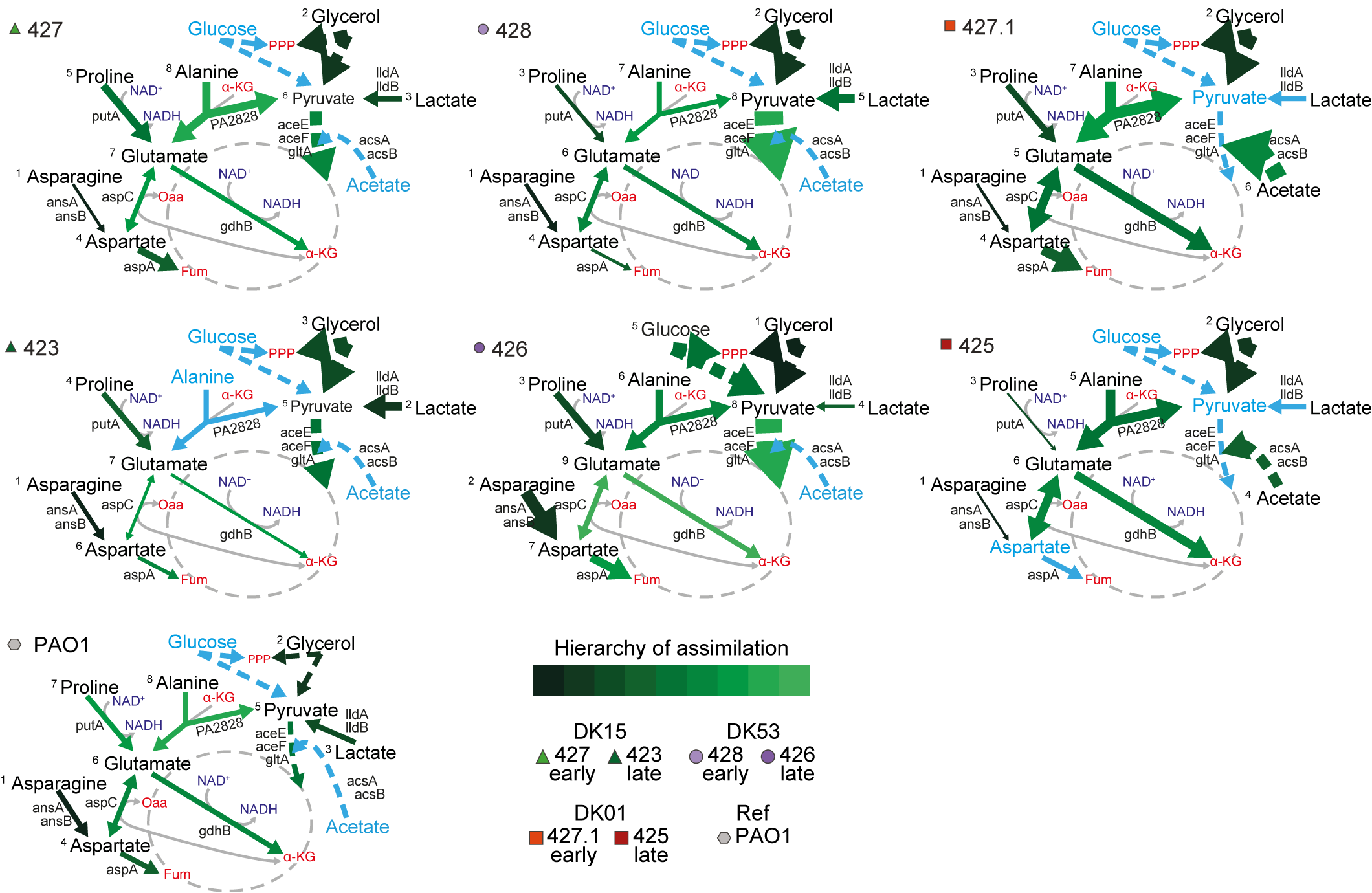

Supplement: FIG S5 [file mbo001183819sf5.tif]

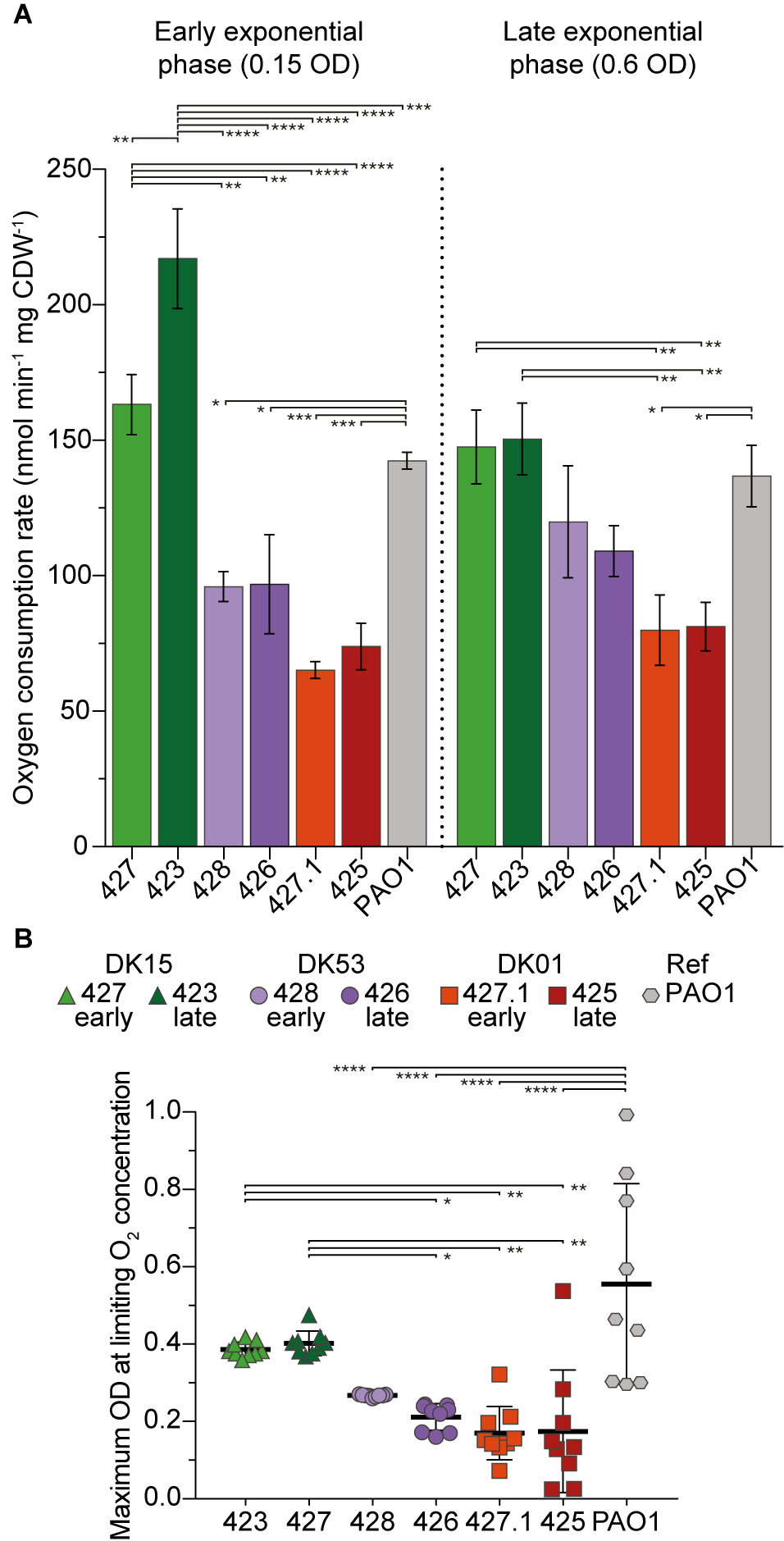

Supplement: FIG S6 [file mbo001183819sf6.tif]

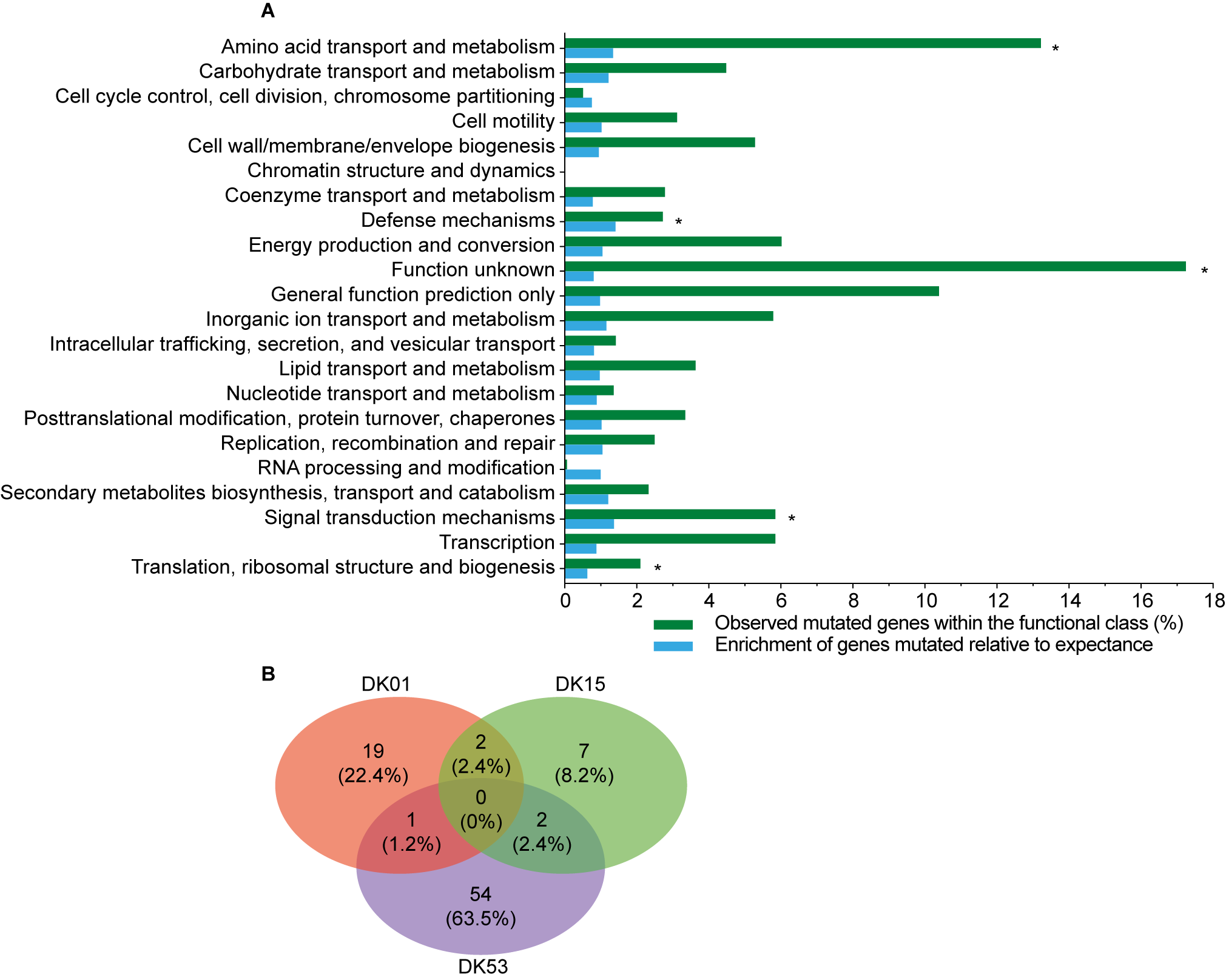

Supplement: FIG S7 [file mbo001183819sf7.tif]
