# Supplementary material for: Convergent Metabolic Specialization through Distinct Evolutionary Paths in Pseudomonas aeruginosa
Source: mBio. 2018 Apr 10;9(2):e00269-18. doi: 10.1128/mBio.00269-18 (PMC5893872; doi:10.1128/mBio.00269-18)
Supplement: TEXT S1 [file mbo001183819s1.pdf]

Materials and Methods

Bacterial strains and media

P. aeruginosa clinical isolates were sampled and identified from sputum samples of the patient P36F2 attending the Copenhagen Cystic Fibrosis Center at the University Hospital, Rigshospitalet, Copenhagen, Denmark (1). Analyses of the bacterial isolates were approved by the local ethics committee of the Capital Region of Denmark (Region Hovedstaden; registration numbers H-1-2013-032). The *P. aeruginosa* laboratory strains PAO1 and PA14 were used as references. Growth kinetics were recorded by measuring the turbidimetry at 630 nm of cultures in 96-well microtitre plates using an ELx808 Absorbance Reader (BioTek Instruments, Winooski, VT, USA). Cells were grown at 37°C with moderate shaking in the rich media LB (2), Artificial Sputum Medium (ASM) (3) and Synthetic CF Sputum Medium (SCFM) (4), or in M9 minimal salt medium (5) with trace elements (6) supplemented with either 20 mM glucose, 20 mM succinate or 20 mM lactate. Growth at limiting oxygen concentration was performed in 96-well microtiter plates with gas impermeable sealing film to avoid oxygen exchange.

Antibiotic sensitivity tests

Minimum inhibitory concentrations (MICs) of 8 different antibiotics were measured by E-test (Liofilchem®, Roseto degli Abruzzi, Italy) according to the manufacturer's guidelines.

Time-resolved exo-metabolome analysis

Dynamic exo-metabolome analyses were performed for isolates 427, 423, 428, 426, 427.1 and 425 and for the reference strain PAO1. A single colony of each isolate from a fresh LB agar plate was inoculated in LB medium and grown overnight at 37ºC. This pre-culture was

used to inoculate, at a turbidity (A_{600}) of 0.05, a 250-ml flask containing 100 ml of fresh pre-warmed LB, and cells were grown at 37ºC at 150 rpm for 24 hours. Throughout growth, 1 ml aliquots were sampled and the A_{600} was scored. Samples were harvested after 0, 1, 2, 3, 4, 5.5, 7, 8.5, 10, 12, 14, 24 hours for isolates 427 and 423 and PAO1 reference strain; 0, 1, 2, 3, 4, 5, 6, 7 (isolate 426) or 7.5 (isolate 428), 9, 10.5, 12, 14, and 24 hours for isolates 428 and 426; and after 0, 1.5, 3, 4.5, 6, 7.5, 9, 10.5, 12, 14, 16, and 24 hours for isolates 427.1 and 425. About 0.95 ml of cell suspension was centrifuged at 21,000 x *g* for 7 minutes at 4°C and the supernatant (0.9 ml) was collected and stored at -80°C for further analysis. For each isolate, three independent biological replicates were analyzed. The same batch of LB was used for all experiments.

Quantification of amino acids, organic acids and sugars

The EZ:faast amino acid analysis kit (Phenomenex, USA) was used for sample preparation and analysis, according to the provided protocol. Briefly, 25 µL of culture supernatant, 100 µL of mQ water and the internal standard were mixed. The solution was pulled into a solid phase extraction (SPE) sorbent tip. Then, 200 µL of washing solvent was pulled through the SPE sorbent tip, followed by 200 µL of elution solvent. The sorbent material was ejected and this step repeated until no sorbent material was left in the tip. Fifty microliters of derivatizing reagent were added, and the solution mixed and incubated for 1 minute. After re-mixing the emulsion, 100 µL of organic reagent were added and the mixture was incubated for 1 min. The upper organic layer was collected and analyzed using a GC-FID (Thermo Fisher Scientific, USA), as described in the provided protocol using a split of 15 and an injection volume of 2 µL. A high pressure liquid chromatography system coupled to a variable wavelength and refractive index detector (HPLC-UV-RI, Ultimate 3000, Dionex,

USA) was used for measurements of glucose, glycerol, pyruvate, acetate, succinate, lactate and formate concentrations. Separation was performed using a Aminex HPX-87H column (Bio-Rad, Sundbyberg, Sweden) held at 45°C. Mobile phase was 5 mM sulfuric acid and pumped isocratically with a flow of 0.6 mL/min.

Non-linear fitting of metabolite concentration

Metabolite concentration was modelled over time or cellular growth (OD) using non-linear regression (7). The concentration of each metabolite was fitted to a four-parameter sigmoid curve ($y = D + A/[1 + e\text{^}[-[x - B]/C]]$) where *B* is the "half-life" parameter, indicated as "t_{50}" or "OD_{50}" when the metabolite concentration was modelled over time or cellular growth reported as A_{600}, respectively. It represents the time or OD at which 50% of a metabolite has been assimilated. To compare hierarchies of assimilation, we used the "OD_{50}" values to avoid errors caused by the differences in the growth rate between the isolates. The *C* parameter represents the "assimilation time" *i.e.*, the time length during which the concentration of a metabolite decreases from 75 to 25% of its initial concentration. Only fittings with goodness of the fit (R square values) higher than 0.9 were considered. The software GraphPad Prism version 7.0a was used for the analysis.

Measurement of oxygen consumption

Four replicates of each isolate were grown as described above in 20% oxygen LB. The decrease in oxygen saturation was measured during growth using a Unisense (Aarhus, Denmark) Clark-type oxygen sensor according to the manufacturer's instructions. Calibration for measurements of oxygen concentrations was performed in water saturated with either air (20% dissolved oxygen) or nitrogen (no dissolved oxygen). To normalize O_2

consumption relative to bacterial growth, oxygen saturation values were fitted to a four-parameter sigmoid model according to growth (A_{600}). During early and late exponential phase at 0.15 and 0.6 OD, respectively, 1 ml of cell suspension was harvested and the respiration rate scored. At 0.6 OD, the cellular suspension was diluted 1:1 with preheated, oxygen-saturated fresh medium. The dry weight of the samples was used as the reference parameter. Oxygen demand during exponential phase was computed as millimoles oxygen consumed per generation per grams of CDW.

Comparative genomics and statistical analyses

Genomic data consisting of SNPs and indels are available for the different isolates (1). We performed Maximum Parsimony (MP) analysis to highlight differences in the mutation distribution between the genomes and to infer the evolutionary history of the bacterial isolates. The bootstrap consensus tree (Fig. 1C) is inferred from 1000 replicates. The MP tree was obtained using the Subtree-Pruning-Regrafting (SPR) algorithm with search level 1 in which the initial trees were obtained by the random addition of sequences (10 replicates). The analysis involved a nucleotide sequence consisting of 2105 concatenated SNP mutations for each of the 26 isolates. Evolutionary analyses were conducted in MEGA7 version 7.0.26 (8). We performed principal component analysis (PCA) to highlight differences in the exo-metabolomes of the cells, by computing the variance of the changes in metabolite concentration over time. To enhance interpretation, the isolates were projected as supplementary classifier which are not included in the calculation of principal components and whose presence does not affect the result. To reduce the complexity of the dynamic exo-metabolome data, we performed PCA using the concentration of each metabolite over the time as independent variables (Fig. S3). Hierarchical cluster analysis was performed

using the Euclidean distance measure and the Ward clustering algorithm on the concentration of each metabolite over the time. In all cases, missing values were estimated using the *k*-nearest neighbour algorithm, and zero values were replaced by the half of the minimum positive value in the data set and finally normalized by log (10) transformation. We used the software JMP version 13.0 for PCA analysis and Hierarchical Clustering. Each biological replicate is represented in the graphs. The growth rates were scored by fitting an exponential curve to the OD data recorded for the three independent biological replicates. The software GraphPad Prism version 7.0a was used for the analysis. In all cases, the goodness of the fit (R square values) was higher than 0.95. Functional enrichment for COGs categories (9) was performed using the binomial statistical method using the software GraphPad Prism version 7.0a. Auxotrophy overrepresentation test was performed using the PANTHER Classification system (10). Genes carrying synonymous mutations were excluded from the analysis. Pearson correlation of growth rate relative to oxygen assimilation rate and of OD_{50} values was calculated using the software GraphPad Prism version 7.0a and JMP version 13.0, respectively.

Data availability

The data that support the findings of this study are available from the corresponding author upon reasonable request.

Bibliography

1. Marvig RL, Sommer LM, Molin S, Johansen HK. 2014. Convergent evolution and adaptation of Pseudomonas aeruginosa within patients with cystic fibrosis. Nat Genet 47:57–64.
2. Sambrook J, Fritsch EF, Maniatis T. 1989. Molecular Cloning: A Laboratory Manual. Cold Spring Harbor laboratory pressNew York.
3. Kirchner S, Fothergill JL, Wright EA, James CE, Mowat E, Winstanley C. 2012. Use of artificial sputum medium to test antibiotic efficacy against Pseudomonas aeruginosa in conditions more relevant to the cystic fibrosis lung. J Vis Exp e3857.
4. Palmer KL, Aye LM, Whiteley M. 2007. Nutritional Cues Control Pseudomonas aeruginosa Multicellular Behavior in Cystic Fibrosis Sputum. J Bacteriol 189:8079–8087.
5. Sambrook J, Russell DW. 2001. Molecular Cloning: A Laboratory Manual. Cold Spring Harb Lab Press Cold Spring Harb NY 999.
6. Bauchop T, Elsden SR. 1960. The growth of micro-organisms in relation to their energy supply. J Gen Microbiol 23:457–69.
7. La Rosa R, Behrends V, Williams HD, Bundy JG, Rojo F. 2016. Influence of the Crc regulator on the hierarchical use of carbon sources from a complete medium in Pseudomonas. Environ Microbiol 18:807–818.
8. Kumar S, Stecher G, Tamura K. 2016. MEGA7: Molecular Evolutionary Genetics Analysis Version 7.0 for Bigger Datasets. Mol Biol Evol 33:1870–1874.
9. Galperin MY, Makarova KS, Wolf YI, Koonin E V. 2015. Expanded Microbial genome coverage and improved protein family annotation in the COG database. Nucleic

Acids Res 43:D261–D269.

10. Mi H, Huang X, Muruganujan A, Tang H, Mills C, Kang D, Thomas PD. 2017. PANTHER version 11: Expanded annotation data from Gene Ontology and Reactome pathways, and data analysis tool enhancements. Nucleic Acids Res 45:D183–D189.
